# The Identification and Management of Refeeding Syndrome in Inpatient Severely Acutely Malnourished Children Aged 6 to 59 Months in Sub-Saharan African Countries: A Systematic Review and Meta-Analysis

**DOI:** 10.3390/children12091223

**Published:** 2025-09-12

**Authors:** Tshegofatso Mogase, Annette Van Onselen, Nidia Rodriguez-Sanchez, Stuart D. R. Galloway

**Affiliations:** 1 Department of Human Nutrition and Dietetics, School of Health Science, Sefako Makgatho Health Sciences University, Ga-Rankuwa 0208, South Africa; 2 Faculty of Health Sciences and Sport, University of Stirling, Stirling FK9 4LA, UK; nidia.rodriguezsanchez@stir.ac.uk (N.R.-S.); s.d.r.galloway@stir.ac.uk (S.D.R.G.); 3 Department of Life and Consumer Sciences, University of South Africa, Pretoria 0002, South Africa; vonsea@unisa.ac.za

**Keywords:** refeeding syndrome, severe acute malnutrition, children under 5 years, Sub-Saharan Africa, inpatient paediatric care, electrolyte imbalance, nutritional rehabilitation

## Abstract

**Background:** Refeeding syndrome is a potentially fatal complication that occurs in inpatient, severely acutely malnourished children during the early phase of nutritional management. Its early identification and management are critical to preventing adverse outcomes. Addressing refeeding syndrome in inpatient settings is critical in Sub-Saharan Africa, where severe acute malnutrition is common and under-researched. **Objective:** To systematically review and meta-analyse current evidence on the identification and management of refeeding syndrome in hospitalised severely malnourished children (6 to 59 months) in Sub-Saharan Africa. **Methods**: A comprehensive search was conducted across academic databases such as PubMed and the Cochrane Library, from 2010 to 2024. Articles reporting on the identification and management of refeeding syndrome in inpatient children with severe acute malnutrition in Sub-Saharan Africa were included. Data extractions were performed by two reviewers using Rayyan software. A meta-analysis of proportions was conducted using STATA 19. **Results**: Nine studies were included. The identification and management of refeeding syndrome were impacted by the lack of a standardised definition. Significant heterogeneity (Q = 27.17, *p* < 0.001) was observed, indicating a significant variation in the prevalence rates ranging from 8.7% to 34.8%. Management strategies varied; most studies adhered to World Health Organisation guidelines for severe acute malnutrition but lacked specific protocols for refeeding syndrome. **Conclusions**: Evidence highlights the need for standardised, evidence-based and context-specific protocols for refeeding syndrome in children with severe acute malnutrition. Early screening, electrolyte monitoring, and cautious feeding remain important, although current evidence is of low certainty. Future prospective studies are needed to develop effective management strategies.

## 1. Introduction

Refeeding syndrome (RFS) remains a poorly understood yet potentially fatal complication in the nutritional rehabilitation of severely acutely malnourished (SAM) children, particularly in inpatient settings [1,2]. Despite recognising RFS as a significant clinical challenge, there has been limited research on its occurrence, diagnosis, and management in Sub-Saharan African countries, where the burden of SAM is highest [3]. Historically, it is accompanied by a range of metabolic and clinical changes that occur when a patient who is malnourished receives aggressive nutritional rehabilitation [4,5]. If no immediate medical care is given to children who are experiencing a fatal change in fluids and electrolytes, then the outcome can include sudden death. The reason for the little to no evidence available in the literature regarding RFS in children with SAM is due to the extremely morbid consequences of starvation and RFS [6]. In addition, it is challenging to study critically ill patients with RFS, especially in younger children with SAM, due to its multifactorial nature. This includes hypokalaemia, hypomagnesemia, thiamine deficiency, abnormal sodium and fluid balance, and changes in glucose, protein, and fat metabolism [5,7,8].

Available studies have shown many ways in which the RFS has been defined. It has been two decades, and still, there is no universal agreement on the definition of RFS [9]. However, in 2020, American Society for Parenteral and Enteral Nutrition (ASPEN) published a consensus recommendation for the definition of RFS which states, “a measurable reduction in levels of one or any combination of phosphorus, potassium, and/or magnesium, or the manifestation of thiamine deficiency, developing shortly (hours or days) after the initiation of calorie provision to an individual who has been exposed to a substantial period of undernourishment” [4]. Due to the lack of a standardised definition, this has also affected the diagnosis, also referred to as identification, management, and monitoring of RFS, particularly in children diagnosed with SAM [1,4,10]. Furthermore, it is difficult to establish the actual frequency of RFS in children with SAM. The difficulty in establishing the frequency and/or the prevalence of RFS is partly due to the lack of a precise definition of the condition and its numerous potential clinical manifestations, and due to the lack of research in this population [3,6].

RFS was first described over 80 years ago [11,12]. It has since been well described and documented in literature in terms of aetiology and pathophysiology, but the challenge remains in defining and, consequently, understanding epidemiology. It remains frequently forgotten and undiagnosed, especially in children with SAM, by healthcare professionals upon admission [6,13]. The discrepancy between changes in homeostatic mechanisms and clinical symptoms makes it challenging to accurately define, diagnose, and/or identify refeeding syndrome [6,14]. Some researchers recommend basing the diagnosis on when clinical symptoms first appear. However, many fluid and electrolyte changes occur without any symptoms, and early recognition and the right treatment can reduce the risk of clinical deterioration [5]. Although children diagnosed with SAM are among the population at high risk of developing RFS, research has shown that not all develop RFS. Therefore, a key question is ‘what factors must be monitored in patients with SAM that could lead to the development of RFS?’ Early detection leading to preventative measures is key in tackling unfavourable outcomes. Therefore, there is a need to understand how RFS is identified and effectively managed in children with SAM, who are known to be a high-risk population. The systematic review aimed to explore the literature on how RFS is identified and managed in hospitalised children with SAM aged 6 to 59 months in Sub-Saharan African countries. To increase awareness in this growing area and propose the development of refined guidelines that minimise or prevent the occurrence of RFS in children hospitalised due to SAM. Thus, the primary objectives of this systematic review article were:

(a) To critically assess the existing literature for the identification of RFS in severely acutely malnourished children aged 6 to 59 months in inpatient settings across Sub-Saharan African countries.
(b) To critically assess the existing guidelines for the management of RFS in severely acutely malnourished children aged 6 to 59 months in inpatient settings across Sub-Saharan African countries.
(c) To critically assess the effectiveness of RFS management guidelines for severely acutely malnourished children aged 6 to 59 months in inpatient settings across Sub-Saharan African countries. Additionally, the review will evaluate how well current guidelines identify children at risk of developing RFS within this severely malnourished group and will examine the key factors contributing to this risk.

## 2. Materials and Methods

### 2.1. Protocol Design and Registration

This is a systematic review, and a meta-analysis was conducted following PRISMA 2020 guidelines [15] between January 2024 and September 2024, and with an additional updated search at the beginning of November 2024. It was registered on PROSPERO (registration number CRD42023491567) in December 2023 to minimise duplication of the same reviews and enhance transparency. The reporting of the findings of this systematic review was developed using the Preferred Reporting Items for Systematic Reviews and Meta-analyses Protocol (PRISMA-P) [16,17].

### 2.2. Study Eligibility Criteria

Inclusion Criteria PICO FRAMEWORK:

- Population (inpatient or hospitalised children aged 6 to 59 months with severe acute malnutrition in Sub-Saharan Africa. This age group is chosen because it represents the population at the highest risk of SAM and associated complications such as RFS. One study included children up to 13 years; however, we retained it because most participants were under 59 months, and subgroup data were not available.
- Intervention (identification and management of refeeding syndrome and nutritional rehabilitation strategies).
- Outcome (incidence of refeeding syndrome or prevalence of refeeding syndrome complications, nutritional recovery rates and mortality rates).
- Study Types (randomised controlled trials, cohort studies, case studies).
- Time Frame (studies published between 2010 and 2024).

Exclusion Criteria:

- Studies outside the specified region or population.
- Non-peer-reviewed articles, letters, and editorials.

### 2.3. Information Source and Search Strategy

A comprehensive search was conducted across PubMed, CINAHL (EBSCO), MEDLINE Ultimate, Web of Science, ScienceDirect, Cochrane Library, and Google Scholar to identify studies published in English between 2010 and 2024. Key search terms included “refeeding syndrome”, “severe acute malnutrition”, “inpatient children”, “Sub-Saharan African countries”, and “hospitalised children”. The detailed Boolean search strings for each database are presented in Appendix A. Minor variations in Boolean operators and synonyms were applied across databases to optimise the retrieval of relevant articles.

### 2.4. Article Screening and Data Extraction

A search of computerised academic databases for all studies published in English from 2010 to 2024 was conducted by two authors (TM and AV), and training was provided before to ensure consistency. The two authors (TM and AV) independently extracted data from all major academic databases and compared their findings, which were imported into the Rayyan software for a systematic review. The data extraction was conducted in two phases: the first phase involved screening and selecting studies based on the titles and abstracts that met the inclusion criteria, and the second phase included screening the full text of the selected eligible studies.

From each included study, we extracted the following information:

- Authors, year, country, and study type;
- Age of the participants, the sample size, and sex;
- Definition of RFS used;
- Time of diagnosis/occurrence;
- Identification and management of RFS;
- Incidence of refeeding syndrome or prevalence of refeeding syndrome;
- Complications of refeeding syndrome upon admission;
- Mortality rates;
- Effectiveness of Guidelines (the WHO protocol aims for less than a 5% mortality rate).

### 2.5. Risk of Bias Assessment

The methodological quality of included studies was assessed using the ROBINS-I tool [18]. This tool evaluates seven domains: confounding, selection of participants, classification of interventions, deviations from intended interventions, missing data, measurement of outcomes, and selection of reported results. Each domain was rated as low, moderate, serious, or critical risk of bias, and an overall judgment was derived for each study. Results are presented in Supplementary Table S1. Risk of bias assessment using ROBINS-I indicated that most included studies were at moderate to serious risk of bias, mainly due to potential confounding, incomplete outcome data, and variability in the classification of RFS. Two studies were judged to be at critical risk of bias due to substantial concerns in multiple domains.

#### Certainty of Evidence

The certainty of evidence for each outcome was assessed using the GRADE approach (Grading of Recommendations, Assessment, Development and Evaluation) [19]. We evaluated study limitations, inconsistency, indirectness, imprecision, and publication bias. Evidence certainty was rated as high, moderate, low, or very low. A GRADE summary of findings table is presented in Supplementary Table S2. The GRADE assessment rated the certainty of the evidence as low to very low across all outcomes. This was primarily due to the observational nature of the included studies, heterogeneity in RFS definitions, small sample sizes, and wide confidence intervals.

### 2.6. Data Synthesis, Data Analysis and Meta-Analysis

The extracted data from the studies included were systematically organised and synthesised to address each of the primary objectives of this systematic review and were entered into a Microsoft Excel worksheet. The key data points included study characteristics, identification methods for RFS, management approaches, risk factors and associated outcomes such as mortality. The extracted data were presented in the evidence table and summarised using descriptive statistics and qualitative analysis due to the heterogeneous nature of the included studies.

A meta-analysis of proportions was performed to estimate the prevalence of RFS. Because hypophosphatemia definitions varied across studies, we prespecified subgroup syntheses by homogeneous definitions. The primary model was a logistic generalised linear mixed model (GLMM) with a random intercept for study (binomial variance, logit link), reporting pooled prevalence with 95% CIs and 95% prediction intervals. Heterogeneity was quantified with τ^2^ and I^2^, and between-subgroup differences were assessed using Q_b. Sensitivity analyses used a two-step random-effects model on the logit scale with REML estimation of τ^2^ and, for comparison, the Freeman–Tukey transformation; a 0.5 continuity correction was applied only to studies with 0%/100% events in the two-step model (none required for GLMM). Individual-study CIs used Clopper–Pearson. For single-study subgroups, we reported the study estimate (no pooling). We explored whether definition type explained heterogeneity using random-effects meta-regression and inspected funnel plots with Egger’s test for small-study effects (recognising low power with few studies). As an exploratory, non-causal comparison, Kruskal–Wallis tests contrasted mortality across reported management approaches. Analyses were conducted in Stata 19.

## 3. Results

### Research Studies Identified Through the Search Strategy

**Figure 1:**
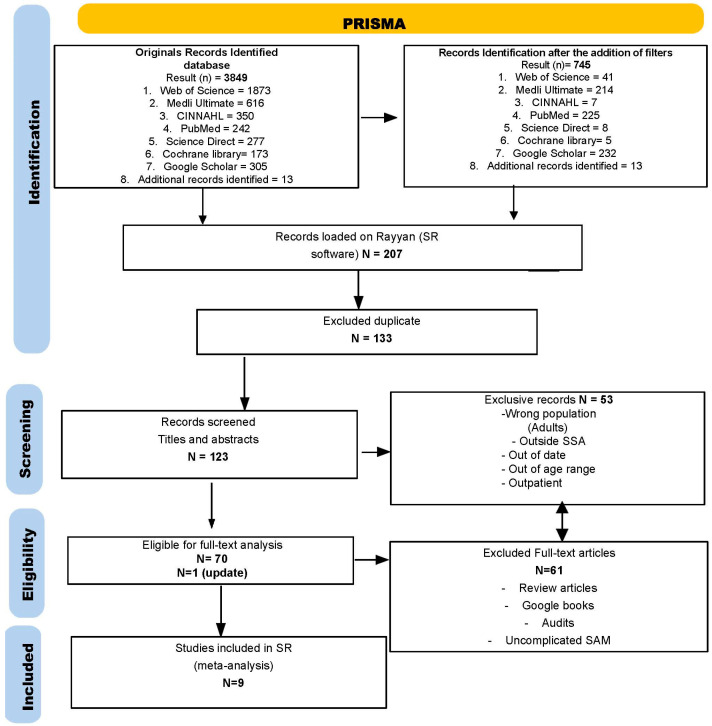
PRISMA flow diagram showing the number of studies screened, included, and excluded.

This systematic review identified 3849 titles and abstracts of potentially eligible studies through academic database searches. After applying filters, 745 records were identified, mainly related to geography (Sub-Saharan African countries) and timeframe (2010–2024). Only 207 were then imported into the Rayan software for systematic review. After removing duplicates, 123 records were screened, and 70 full texts were assessed for eligibility. A total of 114 articles were excluded because they concerned the wrong population (Adults), were outside the geographical scope (Sub-Saharan Africa), were outside the specified date range, focused on outpatient management, did not report the intended outcome, or for other reasons, as illustrated in Figure 1. One additional study was included following an updated search at the beginning of November 2024. Ultimately, nine studies were included in the analysis, as shown in Figure 1 and Table 1, based on the Preferred Reporting Items for Systematic Reviews and Meta-Analyses (PRISMA) checklist. The detailed characteristics of these nine studies are presented in Table 1.

Among the nine studies, seven reported the definition of RFS as shown in Table 2. Definitions were highly heterogeneous among studies, with some only relying on electrolyte disturbances, with different cutoffs, and others also integrating clinical signs and symptoms into the definition. Some studies had hypophosphatemia, as a cutoff or a relative decrease from baseline, as part of the definition. Also, Marik’s definition [28] was reused by most studies, and slightly adapted in two other studies. These definitions are thus the most commonly used.

The prevalence of RFS was highly dependent on the definition used in this SAM population. As shown in Table 2, only 4 of 9 studies reported on the prevalence of RFS. It ranged from 8.7 to 34.8%. Two (2) studies only reported that the electrolyte levels were low, particularly low phosphate; no value was reported.

To address objective one, a meta-analysis of prevalence was conducted to determine the proportion of cases identified using different electrolyte thresholds for diagnosing RFS across studies. The results presented in Table 3 indicated significant variation in the methods used for identifying RFS across the studies reviewed. Particularly, more studies than others reported different levels of hypophosphatemia threshold as the definition of RFS, followed by studies that reported specific drops in electrolyte levels, while one study reported clinical signs such as metabolic derangement as the definition of RFS.

Using a random-effects model on the logit scale with back-transformation to proportions, the results shown in Table 4 indicate that the proportions of RFS across four studies included in the meta-analysis varied significantly, ranging from 8.7% to 34.8%. The overall pooled proportion effect size was 14.0% (95% CI [5.7%, 30.4%]), with a wide 95% prediction interval of [0.9%, 85.7%], indicating substantial dispersion expected in new settings. A significant prevalence (z = −4.13, *p* < 0.001) was also found from the test of theta, suggesting that the pooled estimate was significantly different from zero. However, the test of homogeneity indicates significant heterogeneity (Q = 24.97, *p* < 0.001), reflecting considerable variation in the proportions observed across the different studies in the model.

Three subgroup analyses explored how RFS prevalence changed under consistent hypophosphatemia definitions (Table 5, Table 6 and Table 7). For the subgroup defined by a relative fall in serum phosphate ≥0.3 mmol/L from baseline [20,27], the pooled prevalence was 27.5% (95% CI 16.3–42.5%), with evidence of between-study heterogeneity (Q = 6.13, df = 1, *p* = 0.013). With only two studies, a reliable prediction interval could not be calculated. For subgroups represented by a single study, we report individual study estimates rather than pooled effects: Mbethe et al. [3] (absolute threshold < 1.0 mmol/L) observed 15.4% (95% CI 9.6–23.6%), and Heydenrych et al. [21] (threshold drop ≥ 0.16 mmol/L to <0.65 mmol/L) observed 8.7% (95% CI 4.9–15.1%). Overall, these analyses demonstrate that prevalence varies systematically with the definition employed, highlighting the importance of standardised diagnostic criteria.

**Figure 2:**
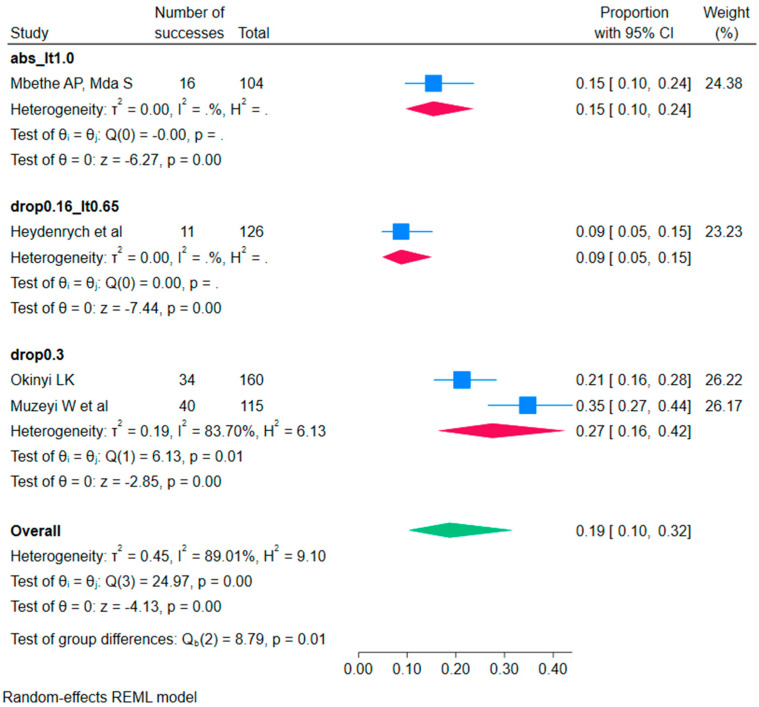
Forest plot of subgroup random-effects meta-analysis of refeeding syndrome prevalence, stratified by homogeneous hypophosphatemia definitions [3,20,21,27].

The results presented in Figure 2 show a subgroup random-effects meta-analysis stratified by homogeneous hypophosphatemia definitions. For the absolute threshold < 1.0 mmol/L subgroup (abs_lt1.0; [3]; k = 1), the study estimate was 0.15 (95% CI 0.10–0.24). For drop ≥ 0.16 to <0.65 mmol/L ([21]; k = 1), the estimate was 0.09 (0.05–0.15). For the relative fall ≥ 0.3 mmol/L from baseline subgroup ([20]; k = 2), the pooled prevalence was 0.27 (0.16–0.42) with substantial heterogeneity (I^2^ = 83.7%, Q (1) =6.13, *p* = 0.01). When all definitions were combined (shown for reference only), the overall random-effects prevalence was 0.19 (0.10–0.32) with high heterogeneity (I^2^ = 89.0%, Q (3) = 24.97, *p* < 0.001); the test of subgroup differences was significant (Qb (2) = 8.79, *p* = 0.01), indicating that prevalence varies by the definition used.

In the random-effects meta-regression of logit-transformed prevalence (Table 8), definition type was entered as the moderator. The model explained 56.46% of the between-study variance (R^2^ analog), yet substantial residual heterogeneity remained (τ^2^ = 0.19; I^2^ = 83.70%). The coefficient for the relative phosphate drop ≥ 0.3 mmol/L definition was positive (β = 0.735 on the logit scale), indicating a higher prevalence relative to the reference definition; however, with few studies, this effect did not reach statistical significance at α = 0.05. The omnibus test of residual heterogeneity was significant (Q_res = 6.13, *p* = 0.013), showing that important variability remains unexplained by the moderator.

**Figure 3:**
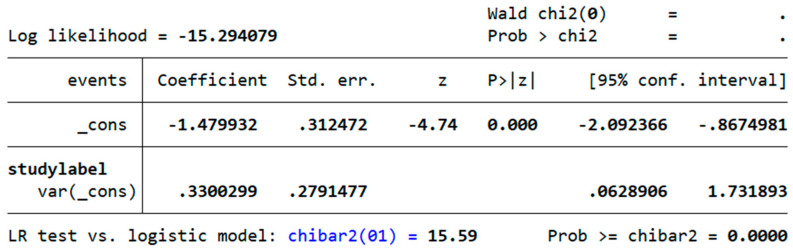
Sensitivity analysis of RFS prevalence using a logistic mixed-effects model with a random intercept for study.

A sensitivity analysis used a GLMM with a random intercept for the study to account for between-study variability, as shown in Figure 3. The intercept was statistically significant (β = −1.48, SE 0.31, z = −4.74, *p* < 0.001, 95% CI −2.09 to −0.87), corresponding to an average prevalence of hypophosphatemia of ≈0.19 on the probability scale (95% CI 0.11–0.30). The estimated between-study variance was σ^2^ᵤ ≈ 0.33 (SE 0.28, 95% CI 0.063–1.732), indicating residual heterogeneity of modest-to-moderate magnitude. A likelihood-ratio test favoured the mixed model over an ordinary logistic model (chibar^2^(1) = 15.59, *p* < 0.001). Overall, the GLMM results align with the primary analyses and support their robustness, while leveraging binomial variance and avoiding continuity corrections for extreme proportions.

The results presented in Table 9 summarise the identification methods, management strategies, and outcomes (mortality rate). In terms of identification, most studies used serum phosphate, potassium, and magnesium levels to diagnose RFS, with variations in specific thresholds. For management, they used the WHO guidelines, which commonly referred to cautious feeding and electrolyte monitoring to prevent complications. Some studies specified the use of therapeutic diets like F-75 and F-100 feeding formulas and electrolyte supplementation, with varying success rates in terms of mortality. One study indicated the lowest mortality rate of 3% among children with RFS, while another reported as high as 18.2% mortality. The mortality data highlight the potential severity of RFS and the importance of early and tailored interventions. Standardised management protocols are crucial for achieving improved patient outcomes.

To address objective 2, a meta-analysis of the various management strategies reported in the studies (e.g., feeding protocols and electrolyte supplementation) was conducted using proportion estimations. The results are presented in Table 10. A slight variation in RFS management guidelines was observed across the studies. These guidelines include electrolyte supplementation and monitoring, feeding protocols, and adherence to WHO guidelines for SAM.

To address objective three, the mortality rate associated with the management strategies reported in the studies was first analysed using the Freeman–Tukey transformed proportions effect size. The results presented in Table 11 showed that the pooled effect size (theta) for mortality across the studies included in the meta-analysis was 0.488 (95% CI: 0.295–0.681). The test of theta indicated statistical significance (z = 2.68, *p* < 0.01). However, the test of homogeneity showed no significant heterogeneity (Q = 3.26, *p* > 0.05), suggesting similarity in the reported mortality rates across studies.

**Figure 4:**
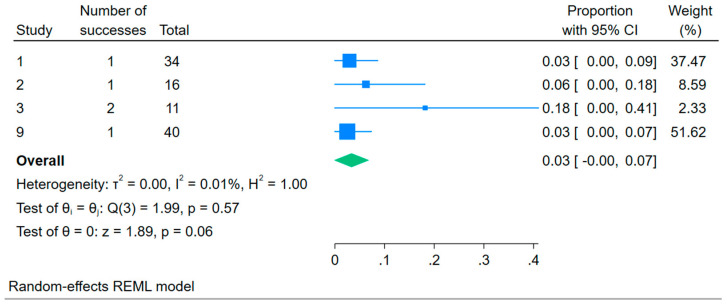
Forest plot showing the overall mortality rate associated with management strategies for children with refeeding syndrome.

The results presented in Figure 4 show that the overall mortality rate associated with management strategies for children with RFS is 0.03 (95% CI: 0.00–0.07). This may indicate that the management strategies are associated with low mortality rates. The forest plot also shows consistency in the reported mortality rates across the reviewed studies.

Finally, a Kruskal–Wallis test was conducted to compare mortality rates based on different management approaches reported in the studies assessing their effectiveness. The results indicated a significant difference in mortality rates across management strategies, particularly between the WHO guidelines for SAM and electrolyte supplementation with monitoring. The findings suggest that applying the WHO guideline for SAM to children with RFS results in the best outcomes, as indicated by the lowest mortality rates, as shown in Table 12.

The results presented in Table 13 outline commonly observed risk factors and complications, including diarrhoea, vomiting, hypophosphatemia, hypomagnesaemia, and hypokalaemia. Respiratory infections, anaemia, and shock were also frequent, suggesting that RFS in malnourished children can lead to severe, multifaceted health issues. Additionally, associated conditions such as HIV, dehydration, and sepsis were commonly reported comorbidities, further exacerbating the vulnerability of these children. The consistency in complications emphasises the systemic nature of RFS and highlights the need for comprehensive monitoring beyond electrolyte levels alone.

**Figure 5:**
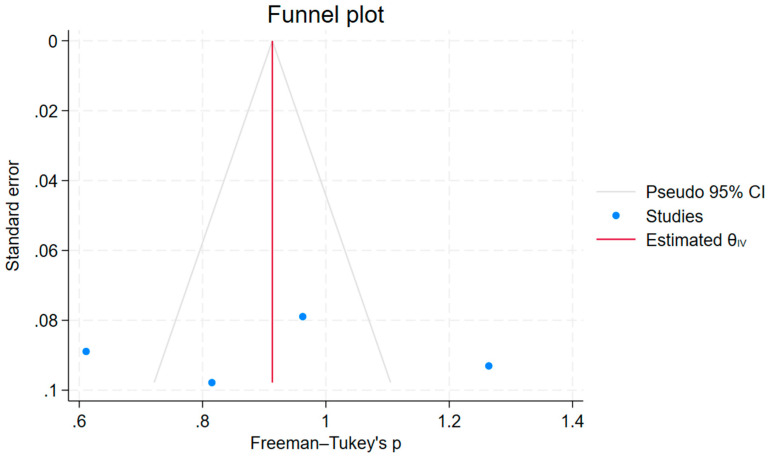
Funnel Plot for Assessment of Publication Bias in Included Studies.

**Figure 6:**
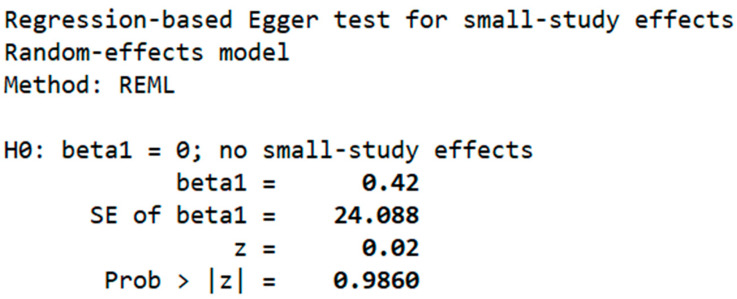
Egger’s regression test for small-study effects (random-effects, REML).

The possibility of publication bias in the studies included in the meta-analysis was explored by observing the distribution of the studies on the funnel plot, as shown in Figure 5. The observed funnel plot shows a symmetrical distribution pattern, suggesting no significant publication bias. This implies that it was appropriate to make a comparison of the proportion from each study to the pooled proportion. Similarly, the Egger test was conducted to assess small-study effects. The results of the Regression-based Egger test in Figure 6 were not significant (*p* > 0.05), suggesting no publication bias.

## 4. Discussion

This systematic review with meta-analysis examined several studies on the identification and management of RFS in hospitalised children aged 6 to 59 months with SAM in Sub-Saharan African countries. According to this review, the key findings highlight that RFS identification and reported prevalence vary primarily depending on the definition of hypophosphatemia applied. When pooled with a random-effects logit model, the overall RFS prevalence was 14% (95% CI 5.7–30.4%). However, the 95% prediction interval (0.9% to 85.7%) underscores the wide range expected in new cohorts. Subgroup re-meta-analyses confirm that stricter or relative drop definitions yield different prevalence bands (≈9% to ≈28%), emphasising the clinical importance of adopting homogeneous criteria when synthesising evidence or planning services. This emphasises the critical need for standardised protocols targeted at this vulnerable population. Furthermore, the results suggest that the lack of a standardised definition and protocols across different studies may impact clinical practice and outcomes.

### 4.1. Identification of Refeeding Syndrome

The results show that all the reviewed studies have different definitions and methods of diagnosis for RFS. Seven of the nine studies relied on electrolyte abnormalities such as hypophosphatemia, hypokalaemia, and hypomagnesaemia to define RFS; however, the threshold values of electrolytes varied significantly. This affected the reported prevalence of RFS, which ranged from 8.7% to 34.8%. For example, studies conducted in East Africa (Kenya and Uganda) reported higher prevalence rates of RFS compared to studies from Southern Africa. The results showed that the prevalence of RFS was highly dependent on the definition used. These results are consistent with the observations made by Friedli et al. (2017) and Cioffi et al. (2021) [13,29], who also reported a lack of standardised definition, consequently varying prevalence of RFS, but reported a slightly higher prevalence. Regional differences were also observed in the types of clinical manifestations reported. East African studies more often described hypophosphatemia and delayed feeding tolerance, whereas Southern African studies highlighted oedematous malnutrition and associated electrolyte disturbances such as hyponatremia and hypokalaemia. Evidence suggests that differences in dietary composition, high prevalence of comorbid conditions such as HIV and tuberculosis [30], and limited monitoring infrastructure in resource-limited settings may contribute to regional variation in clinical manifestations and outcomes. These findings underscore the importance of considering local determinants when developing specific clinical guidelines for the identification and management of RFS.

Moreover, the disparity in the prevalence emphasises how difficult it can be to correctly diagnose RFS in hospitalised children with SAM because some studies have focused solely on electrolyte disturbances [3,20,21,26,27] while others also included clinical symptoms [22]. Most studies used hypophosphatemia as a defining surrogate marker of RFS, which aligns with existing literature [31]. However, it also raises concerns about underdiagnosis when other electrolyte imbalances are not taken into account. Furthermore, there were variations in the timing of RFS diagnosis; some studies reported cases at admission, while others reported cases between 48 and 13 days after refeeding. Electrolyte abnormalities detected at admission may reflect the underlying pathophysiology of SAM and/or associated illnesses such as diarrhoea rather than RFS, which by definition emerges after the initiation of feeding. Accordingly, baseline biochemical markers should be interpreted as risk stratification, whereas post-refeeding declines in phosphate (and/or potassium/magnesium), particularly within the first 24 to 72 h of nutritional rehabilitation, are more consistent with incident RFS. Inconsistent diagnostic timelines make early detection of RFS more difficult, highlighting the critical need for a standardised methodology in clinical settings.

### 4.2. Proposal to Refine the Definition for Refeeding Syndrome in Inpatient Severe Acute Malnutrition

Our subgroup re-meta-analysis showed that reported RFS prevalence varies systematically with how hypophosphatemia is defined. To improve comparability across studies and support clinical decision-making in SAM, we propose a harmonised, operational definition that standardises (i) the time window, (ii) biochemical anchors, and (iii) severity grading, while remaining feasible in low-resource settings. Proposed definition: RFS in inpatient children with SAM is new or worsening hypophosphatemia occurring within 72 h of starting feeds or ≥50% escalation of energy provision, defined by either (a) serum phosphate below the age-appropriate lower limit of normal (or <0.65–0.80 mmol/L where local limits are unavailable) or (b) a ≥20% fall from baseline phosphate; accompanying hypokalaemia and/or hypomagnesemia and compatible cardiac, respiratory, or neurologic signs increase diagnostic certainty and grade severity, but are not required for diagnosis.

### 4.3. Management Strategies and Effectiveness

The review found descriptive variation in management strategies for RFS in children with SAM across studies, although most adhered to WHO guidelines for SAM treatment. Nonetheless, statistical testing (Kruskal–Wallis) did not demonstrate significant differences between strategies. The most frequently reported methods of management were cautious feeding using F-75 and F-100 formulas (33.3%), followed by electrolyte supplementation and monitoring (22.2%), and gradual caloric increase in line with the WHO guidelines for SAM at 22.2%. However, inconsistencies in monitoring electrolyte levels and adjusting treatment protocols were noted across studies. Even though WHO guidelines are the globally accepted standard for managing SAM, the heterogeneity in management approaches suggests that there are no unified procedures for RFS in inpatient settings. Therefore, while management approaches vary, these findings should be interpreted with caution, and further studies are needed to confirm whether such differences translate into clinical outcome variations.

Mortality rates were used to evaluate the effectiveness of these management strategies. The analysis showed a significant risk associated with RFS, as demonstrated by the pooled mortality rate among children who developed RFS, which ranged from 3% to 18.2%. In contrast to RFS prevalence, mortality rates were uniform across studies, demonstrating no significant heterogeneity.

Studies that used gradual nutritional rehabilitation and close electrolyte monitoring, which aligns with the WHO recommendations, had the lowest mortality rates, making them the most effective approach. However, despite adherence to WHO guidelines, the variation in outcomes suggests the need for further investigation into the specific management protocols employed in different contexts, as well as the potential need for tailored interventions based on local resources and practices that specifically address RFS risks in children with SAM.

### 4.4. Risk Factors and Complications

The review also found that diarrhoea, hypophosphatemia, and hypomagnesaemia are common risk factors and outcomes associated with RFS. These findings emphasise the complex nature of RFS and the importance of comprehensive monitoring that extends beyond electrolyte levels. Improving the overall health of these vulnerable groups requires addressing comorbidities such as sepsis, dehydration, and HIV.

### 4.5. Clinical Implications

This review contributes to clinical practice by highlighting the absence of systematic screening and monitoring protocols for RFS in children with SAM across Sub-Saharan Africa. In view of the inconsistent identification and management strategies reported, the findings underscore the importance of prioritising routine monitoring of phosphate, potassium, and magnesium during the first 72 h of stabilisation, alongside the adoption of cautious feeding regimens consistent with WHO recommendations. By synthesising evidence from diverse African contexts, this review identifies key risk markers that are frequently overlooked in clinical settings and provides a foundation for the development of context-specific guidelines. Ultimately, implementing such measures may improve early detection of RFS, reduce complications, and contribute to lowering mortality among hospitalised children with SAM.

### 4.6. Challenges and Limitations

Several challenges in managing RFS in the SAM population have been identified in this review study. The certainty of the evidence identified in this review was generally rated as low to very low according to the GRADE framework. This reflects the predominance of observational study designs, methodological limitations highlighted in the ROBINS-I assessment, and the considerable heterogeneity in definitions, diagnostic thresholds, and reporting of outcomes across studies. In addition, the small number of eligible studies and relatively small sample sizes contributed to imprecision in the pooled estimates. These factors limit the strength of inferences that can be drawn. Furthermore, the absence of a universally accepted definition for RFS complicates the estimation of prevalence. The results highlight the need for more thorough research, including prospective studies and randomised trials, to establish standardised identification and management protocols. Additionally, the clinical manifestation of RFS in children with SAM is further complicated by underlying comorbidities like infections, anaemia, and HIV. Given how these conditions interact, a multidisciplinary strategy comprising paediatricians, dietitians, and infectious disease experts may help patients have improved outcomes.

## 5. Conclusions

This systematic review and meta-analysis highlight the critical gaps in the identification and management of RFS in hospitalised children with SAM in Sub-Saharan Africa. The variation in definitions, diagnostic thresholds, and management strategies was observed, highlighting the urgent need for evidence-based, standardised definitions and recommendations designed explicitly for this high-risk population. In line with our GRADE assessment, the certainty of the evidence was rated as low to very low, which limits the strength of conclusions that can be drawn. Nonetheless, the findings suggest that strengthening routine screening procedures, regular monitoring of electrolytes, and cautious refeeding practices remain critical components of care. Prospective studies should be the focus of future research to assess the efficacy of various management techniques and improve patient outcomes by improving current guidelines. Moreover, there is a great need to strengthen healthcare practitioner training, raise awareness, and create region-specific standard protocols in the identification and management of RFS in hospitalised children with SAM.

## Figures and Tables

**Table 1:** Characteristics of the selected studies.

Author, Year of Publication	Country	Type Study	Age (Month)	Sample Size	Male (*n*)	Female (*n*)
Okinyi LK, 2018 [20]	Kenya	Observational study	6–59	160	51% (83)	49% (77)
Mbethe AP & Mda S, 2017 [3]	South Africa	Prospective observational study	4.4–49.5	104	56% (58)	44% (46)
Heydenrych et al., 2024 [21]	South Africa	Retrospective cohort study	0–59	126	63% (79)	37% (47)
Bandsma et al., 2019 [22]	Kenya and Malawi	Double-blind, randomised controlled trial	6 m–13 yrs	843	NR	46% female
Chatenga H, 2021 [23]	Ghana	Longitudinal observational study design with a quantitative, descriptive comparative	6–59	380 medical records	54%	NER
Hother et al., 2016 [24]	Ethiopia	Prospective observational study	6–59	72	NR	32
Namusoke et al., 2016 [25]	Uganda	Prospective observational study	6–59	120	NR	46 (38%)
Rytter et al., 2017 [26]	Uganda	Prospective cohort study	6–59	120	NR	45 (38%)
Muzeyi et al., 2024 [27]	Uganda	Prospective cohort study	6–59	115	NR	NR

NR denotes Not reported. NER denotes Not Explicitly Reported.

**Table 2:** Definition, prevalence, and timely occurrence of Refeeding Syndrome.

Author, Year of Publication	Country	Definition of Refeeding Syndrome	Prevalence of Refeeding Syndrome	Time of Diagnosis/Occurrence
Okinyi LK, 2018 [20]	Kenya	Significant electrolyte imbalances occurring after feeding are initiated, particularly hypokalaemia and hypophosphatemia.	21% (34 out of 160 children with SAM developed RFS)	At admission and 48 h after feed initiation
Mbethe AP & Mda S, 2017 [3]	South Africa	The hallmark biochemical change of RFS was defined as hypophosphatemia.	15% (16 out of 104 children)	On day 5 of hospitalisation
Heydenrych et al., 2024 [21]	South Africa	Marik et al. define RFS solely as decreased serum phosphate levels after the initiation of MNT.	8.7% (11 out of 126 children developed RFS	Within 5 days of admission and 6–13 days
Bandsma et al., 2019 [22]	Kenya and Malawi	NCR: characterised by hypophosphatemia, hypokalaemia, and hypomagnesemia, which may impair cardiac, pulmonary, and neurological function and can result in (sudden) death.	NR	At admission and on day 3
Chatenga H, 2021 [23]	Ghana	NER: A potentially fatal SAM complication due to a rapid increase in the amount of nutrition given to a child with complicated SAM may also lead to electrolyte disequilibrium, referred to as refeeding syndrome.	NR	Upon admission and the transition phase
Hother et al., 2016 [24]	Ethiopia	NEC: referred to as refeeding hypophosphatemia and hypomagnesemia	NER, but Phosphate and magnesium levels were reported as below normal at admission, with 63 of 68 children (93%) experiencing hypophosphatemia, leading to the conclusion that hypophosphatemia is the primary biochemical hallmark of RFS	Upon admission, at the start of the rehabilitation phase, and at discharge
Namusoke et al., 2016 [25]	Uganda	Change in phosphate levels in the context of refeeding (severe hypophosphatemia).	0% (None developed severe hypophosphatemia)	Upon admission and on Day 2
Rytter et al., 2017 [26]	Uganda	Hypophosphatemia is defined as plasma phosphate concentration < 1.6 mmol/L for children ≤ 12 months and < 1.1 mmol/L for older children.	NER: Low plasma phosphate is associated with increased mortality (14%)	Upon admission and on Day 2
Muzeyi et al., 2024 [27]	Uganda	RFS was defined based on the change in serum phosphorus levels (phosphorus drop of 0.3 mmol/L or more from the baseline value).	34.8% (40 out of 115)	Upon admission and in 48 h

NR denotes Not Reported; NCR denotes No Clear Report. NER denotes Not Explicitly Reported. NEC denotes Not Explicitly Clear. MNT denotes Medical Nutrition Therapy. RFS denotes Refeeding Syndrome.

**Table 3:** Proportion Estimation of Refeeding Syndrome Identification Methods.

	Proportion	Std. Err.	95% CI	
Drop in Electrolyte Levels	0.22	0.14	0.043	0.645
Hypophosphatemia Threshold	0.44	0.17	0.146	0.789
Clinical Signs	0.11	0.10	0.011	0.591
No Explicit Reporting	0.11	0.10	0.011	0.591
Not Reported	0.11	0.10	0.011	0.591

Number of observations = 9.

**Table 4:** Pooled prevalence of refeeding syndrome across included studies (random-effects meta-analysis of proportions).

Study	Country	Proportion	95% CI		% Weight
[20]	Kenya	0.21	0.156	0.283	26.2
[3]	South Africa	0.15	0.096	0.236	24.4
[21]	South Africa	0.09	0.049	0.151	23.2
[27]	Uganda	0.35	0.267	0.439	26.2
Invlogit (theta)		0.14	0.0.57	0.304	

95% prediction interval for invlogit (theta): [0.009, 0.857]. Test of theta = 0: z = −4.13. Prob > |z| = 0.0000. Test of homogeneity: Q = chi2(3) = 24.97. Prob > Q = 0.0000.

**Table 5:** Subgroup prevalence of refeeding syndrome under a homogeneous hypophosphatemia definition (relative fall ≥ 0.3 mmol/L from baseline; k = 2).

Study	Country	Proportion	95% CI		% Weight
Okinyi 2018 [20]	Kenya	0.21	0.156	0.283	50.1
Muzeyi et al. 2024 [27]	Uganda	0.35	0.267	0.439	49.9
Invlogit (theta)		0.28	0.163	0.425	

95% prediction interval for invlogit (theta). Test of theta = 0: z = −2.85. Prob > |z| = 0.0044. Test of homogeneity: Q = chi2(1) = 6.13. Prob > Q = 0.0133.

**Table 6:** Subgroup prevalence of refeeding syndrome under an absolute hypophosphatemia threshold < 1.0 mmol/L (single-study subgroup; k = 1).

Study	Country	Proportion	95% CI		% Weight
Mbethe et al., 2017 [3]	South Africa	0.15	0.096	0.236	100.0
Invlogit (theta)		0.15	0.096	0.236	

95% prediction interval for invlogit (theta). Test of theta = 0: z = −6.27. Prob > |z| = 0.0000. Test of homogeneity: Q = chi2(0) = −0.00.

**Table 7:** Subgroup prevalence of refeeding syndrome under a threshold drop ≥ 0.16 mmol/L to <0.65 mmol/L (single-study subgroup; k = 1).

Study	Country	Proportion	95% CI		% Weight
Heydenrych et al., 2024 [21]	South Africa	0.09	0.049	0.151	100.0
Invlogit (theta)		0.09	0.049	0.151	

95% prediction interval for invlogit (theta). Test of theta = 0: z = −7.44. Prob > |z| = 0.0000. Test of homogeneity: Q = chi2(0) = −0.00.

**Table 8:** Random-effects meta-regression of logit-transformed prevalence by hypophosphatemia definition (definition as moderator).

Predictor (Reference = <1.0 mmol/L)	Coef (Log-odds)	SE	Z	*p*	[95% CI]	
drop0.16_1t0.65	−0.64	0.75	−0.86	0.39	−2.112	0.827
drop0.3	0.73	0.62	1.19	0.24	−0.480	1.949
constant	−1.70	0.52	−3.29	0.001	−2.719	−0.689

Test of residual homogeneity: Q_res = chi2 (1) = 6.13. Prob. Q_res = 0.0133. Residual heterogeneity: tau2 = 0.1943, I^2^ (%) = 83.70, H^2^ = 6.13, R-squared (%) = 56.46, Ward chi2 (2) = 4.96, Prob > chi2 = 0.0835.

**Table 9:** Identification and management of refeeding syndrome, nutritional recovery rates and mortality rates.

Author, Year of Publication	Country	Identification of Refeeding Syndrome	Management of Refeeding Syndrome	Mortality Rate
Okinyi LK, 2018 [20]	Kenya	Electrolyte levels (potassium, phosphorus, magnesium). The diagnosis was based on a drop of >0.3 mmol/L from baseline for either potassium or phosphorus.	Electrolyte supplementation and monitoring were provided. Patients were monitored for over one week.	3% (1 out of 34 children who developed refeeding syndrome died).
Mbethe AP & Mda S, 2017 [3]	South Africa	Electrolyte levels, specifically hypophosphatemia (<1 mmol/L).	Cautious feeding with a low-energy and low-protein diet according to WHO guidelines, starting with the F75 formula, and monitoring electrolytes.	6% among children who developed refeeding syndrome.
Heydenrych et al., 2024 [21]	South Africa	A drop in phosphate levels > 0.16 mmol/L to below 0.65 mmol/L after feeding was initiated.	WHO treatment guidelines for SAM include progressive feeding and careful monitoring of electrolytes.	18.2% of children who developed refeeding syndrome
Bandsma et al., 2019 [22]	Kenya and Malawi	Clinical signs such as metabolic derangements, including hypophosphatemia, hypokalemia, and hypomagnesemia.	By monitoring electrolyte levels and adjusting nutritional intake.	NR
Chatenga H, 2021 [23]	Ghana	NER	NCR: The study focuses on feeding regimens during the transition phase.	NR
Hother et al., 2016 [24]	Ethiopia	NER but at admission: Hypophosphatemia was found based on age-specific reference cut-offs (serum phosphate < 1.45 mmol/L).	Children were fed therapeutic diets (F-75 and F-100) during different phases of treatment.	NR
Namusoke et al., 2016 [25]	Uganda	Plasma phosphate levels were monitored to assess phosphate status.	Using F-75 and F-100 therapeutic formulas, with careful monitoring of phosphate levels during refeeding.	NR
Rytter et al., 2017 [26]	Uganda	Biochemical Marker: Hypophosphatemia (Low Plasma Phosphate), and clinical symptoms such as oedema, were monitored on admission and again at 48 h post-admission.	Gradual increase in energy intake and inclusion of phosphorus in therapeutic formulas (F-75 and F-100).	14% of the sample died;
Muzeyi et al., 2024 [27]	Uganda	By a drop in serum phosphorus of more than 0.3 mmol/L from baseline.	NER, but indicate careful monitoring and gradual transition from F75 to Ready-to-Use Therapeutic Feeds (RUTF) were employed.	2.7% (4 OF 150 Died)

Note: The management strategies presented reflect interventions reported by the included studies. As no universally accepted guidelines for RFS currently exist, most authors relied on WHO recommendations for managing SAM, particularly cautious feeding with F-75 and F-100 formulas and electrolyte monitoring, as preventive or supportive measures against RFS. NR denotes Not Reported. NCR denotes No Clear Report. NER denotes Not Explicitly Reported.

**Table 10:** Proportion Estimation of Existing Guidelines for the Management of Refeeding Syndrome.

	Proportion	Std. Err.	95% CI	
Electrolyte Supplementation and Monitoring	0.22	0.14	0.043	0.645
Feeding Protocols (e.g., F-75, F-100)	0.33	0.16	0.089	0.719
WHO Guidelines for SAM	0.22	0.14	0.043	0.645
No Explicit Reporting	0.11	0.11	0.011	0.591
Not Reported	0.11	0.11	0.011	0.591

**Table 11:** Mortality among children with refeeding syndrome in included studies.

Study	Country	Effect Size	95% CI		% Weight
[20]	Kenya	0.41	0.078	0.745	33.5
[3]	South Africa	0.60	0.113	1.078	16.0
[21]	South Africa	0.94	0.366	1.522	11.2
[27]	Uganda	0.38	0.072	0.687	39.3
theta		0.49	0.295	0.681	

Test of theta = 0: z = 2.68. Prob > |z| = 0.0075. Test of homogeneity: Q = chi2(3) = 3.26. Prob > Q = 0.3530.

**Table 12:** Kruskal–Wallis equality-of-proportions rank test.

	Observation	Rank Sum
Electrolyte Supplementation and Monitoring	1	2.0
WHO Guidelines for SAM	2	7.0
No Explicit Reporting	1	1.0

Chi2(2) = 2.700. Prob = 0.2592.

**Table 13:** Risk factors and complications of refeeding syndrome.

Author, Year of Publication	Country	Type of Study	Risk Factors, Complications of Refeeding Syndrome at Admission and During Treatment
Okinyi LK, 2018 [20]	Kenya	Observational study	Vomiting, diarrhoea.
Mbethe AP & Mda S, 2017 [3]	South Africa	Prospective observational study	Diarrhoea, shock, hypokalaemia, hypocalcaemia, hypomagnesaemia.
Heydenrych et al., 2024 [21]	South Africa	Retrospective cohort study	Hypophosphatemia, hypokalaemia, dehydration, coagulopathy, urinary tract infections, and diarrhoea.
Bandsma et al., 2019 [22]	Kenya and Malawi	Double-blind, randomised controlled trial	Metabolic derangements, specifically hypophosphatemia, hypokalaemia, and hypomagnesemia.
Chatenga H, 2021 [23]	Ghana	longitudinal observational study design with a quantitative, descriptive comparative	Diarrhoea, vomiting, and respiratory infections.
Hother et al., 2016 [24]	Ethiopia	Prospective observational study	Hypophosphatemia and hypomagnesemia.
Namusoke et al., 2016 [25]	Uganda	Prospective observational study	Diarrhoea, hypophosphatemia, cough, fever, rash.
Rytter et al., 2017 [26]	Uganda	Prospective cohort study	Respiratory and circulatory failure, death.
Muzeyi et al., 2024 [27]	Uganda	Prospective cohort study	NER but indicated significant values of oedema, vomiting, and diarrhoea.

## Data Availability

The original data presented in the study are openly available in all the academic databases mentioned in the Section 2.

## Supplementary Materials

The following supporting information can be downloaded at: https://www.mdpi.com/article/10.3390/children12091223/s1, Table S1: Risk of Bias Assessment (ROBINS-I); Table S2: GRADE summary of findings: Identification and management of refeeding syndrome in children with severe acute malnutrition in sub-Saharan Africa.

## Abbreviations

The following abbreviations are used in this manuscript:

SAM	Severe Acute Malnutrition
WHO	World Health Organisation
RFS	Refeeding Syndrome
ASPEN	American Society for Parenteral and Enteral Nutrition
GRADE	Grading of Recommendations, Assessment, Development and Evaluation
GLMM	Generalised Linear Mixed Model

## Appendix A

**Example of search strategy in PubMed**

(“severely acutely malnourished children” OR “inpatient children” OR “children aged 6 to 59 months”) AND (“identification of refeeding syndrome” OR “management of refeeding syndrome” OR “nutritional rehabilitation strategies”) AND (“Sub-Saharan African countries”) AND (“incidence of refeeding syndrome” OR “complications of refeeding syndrome” OR “nutritional recovery rates” filters: 2010/01/01 to 2024/08/08, English, 1 month to 5 years

**Example of the search strategy in EBSCOhost [CINNAHL (EBSCO), MEDLINE Ultimate]**

severe acute malnutrition AND (hospitalised child or pediatric or paediatric or children) OR inpatient children AND 6-59 months AND (sub-Saharan Africa or sub-Saharan Africa or sub sahara or sub-Sahara) AND identification of refeeding syndrome AND management of refeeding syndrome OR nutritional rehabilitation strategies AND (incidence and prevalence of refeeding syndrome) AND (mortality or mortality rate or death or death rate) AND nutritional recovery rate.
